# Adaptive response to starvation in the fish pathogen *Flavobacterium columnare*: cell viability and ultrastructural changes

**DOI:** 10.1186/1471-2180-12-266

**Published:** 2012-11-19

**Authors:** Covadonga R Arias, Stacey LaFrentz, Wenlong Cai, Oscar Olivares-Fuster

**Affiliations:** 1 Department of Fisheries and Allied Aquacultures, Aquatic Microbiology Laboratory, Auburn University, 203 Swingle Hall, Auburn, AL, 36849, USA

**Keywords:** *Flavobacterium columnare*, Starvation, Ultrastructure, Survival, Columnaris disease

## Abstract

**Background:**

The ecology of columnaris disease, caused by *Flavobacterium columnare*, is poorly understood despite the economic losses that this disease inflicts on aquaculture farms worldwide. Currently, the natural reservoir for this pathogen is unknown but limited data have shown its ability to survive in water for extended periods of time. The objective of this study was to describe the ultrastructural changes that *F*. *columnare* cells undergo under starvation conditions. Four genetically distinct strains of this pathogen were monitored for 14 days in media without nutrients. Culturability and cell viability was assessed throughout the study. In addition, cell morphology and ultrastructure was analyzed using light microscopy, scanning electron microscopy, and transmission electron microscopy. Revival of starved cells under different nutrient conditions and the virulence potential of the starved cells were also investigated.

**Results:**

Starvation induced unique and consistent morphological changes in all strains studied. Cells maintained their length and did not transition into a shortened, coccus shape as observed in many other Gram negative bacteria. *Flavobacterium columnare* cells modified their shape by morphing into coiled forms that comprised more than 80% of all the cells after 2 weeks of starvation. Coiled cells remained culturable as determined by using a dilution to extinction strategy. Statistically significant differences in cell viability were found between strains although all were able to survive in absence of nutrients for at least 14 days. In later stages of starvation, an extracellular matrix was observed covering the coiled cells. A difference in growth curves between fresh and starved cultures was evident when cultures were 3-months old but not when cultures were starved for only 1 month. Revival of starved cultures under different nutrients revealed that cells return back to their original elongated rod shape upon encountering nutrients. Challenge experiments shown that starved cells were avirulent for a fish host model.

**Conclusions:**

Specific morphological and ultrastructural changes allowed *F*. *columnare* cells to remain viable under adverse conditions. Those changes were reversed by the addition of nutrients. This bacterium can survive in water without nutrients for extended periods of time although long-term starvation appears to decrease cell fitness and resulted in loss of virulence.

## Background

*Flavobacterium columnare* is a Gram negative bacterium, member of the Cytophaga-Flavobacterium-Bacteroides (CFB) group, and the causative agent of columnaris disease in fish [1]. Columnaris disease affects freshwater fish species around the world and is responsible for major economic losses in catfish and tilapia aquaculture [2-4]. Because of its economic impact, most studies on *F*. *columnare* have focused on the pathogenesis of this bacterium as well as on detection and prevention strategies against the disease [5-7]. In experimental aquaculture settings, columnaris disease can be transmitted by fish to fish contact or through contaminated water [7]. However, the natural reservoir and survival strategies of *F*. *columnare* in the aquatic environment are not well understood. Early studies on survival of *F*. *columnare* in artificial microcosms proved that this bacterium could survive in water for extended periods of time but optimal conditions for survival were inconclusive [8,9]. Fijan [8] reported that *F*. *columnare* survived better in water with high organic matter content while Chowdhury and Wakabayashi [9] showed that *F*. *columnare* cells remained viable without organic nutrients. In a recent study, it was shown that *F*. *columnare* can survive for up to 5 months in either distilled water or lake water leading to the conclusion that this bacterium behaves as an opportunistic pathogen with a saprophytic lifestyle that uses water as natural reservoir [10].

Aquatic bacteria can be subject to rapid changes in nutrient availability and must adapt accordingly in order to survive [11]. In well-studied bacteria, such as *Vibrio* spp. and *Pseudomonas* spp., the first noticeable change in cell structure upon encountering starvation conditions is dwarfing [12]. Cells can undergo a reduction division, which will increase cell numbers with the corresponding reduction in overall cell size, or they can directly reduce their volume. Along with a reduction in size, cells typically become rounder adopting a coccus morphology in what is known as the ‘rounding up’ strategy [13]. In the species *F*. *psychrophilum*, a coldwater fish pathogen, starvation studies have shown the ability of this pathogen to maintain culturability in stream water for at least 19 weeks [14]. However, changes in cell morphology were not as evident as in other Gram negative bacteria. The majority of *F*. *psychrophilum* cells remaining as long and thin bacilli, few showing round enlargements, and in some cases, they adopted a ring-like conformation. The response of *F*. *columnare* to short- and long-term starvation has been studied based on cell culturability [8-10] but characterization on the morphological and physiological changes that accompany this phenomenon have not been investigated in this species. The objective of this study was to assess the potential of *F*. *columnare* to survive under starvation conditions as well as to characterize the ultrastructural changes in cell morphology that accompanies this process.

## Methods

### Bacterial strains

Four previously characterized *F*. *columnare* strains were used in this study representing two of the genomovars described within the species [15,16]. Genomovar I strains included the type strain ATCC 23463, originally isolated from Chinook salmon, and strain ARS-1 recovered from channel catfish. Genomovar II was represented by strains ALG-00-530 and AL-02-036 isolated from channel catfish and largemouth bass, respectively. Virulence between genomovar I and II strains is significantly different in channel catfish. Selected genomovar II strains are highly virulent in channel catfish fingerlings (mortality >90%) while genomovar I strains are less (ARS-1 produces <50% mortality) or not virulent (ATCC 23463) [17]. Bacteria were stored at −80°C as glycerol stocks and routinely cultured on modified Shieh agar (MS) or broth with shaking (125 rpm) at 28±2°C for 24–48 h [18].

### Survival under starvation conditions

Individual colonies from each strain were inoculated into 4 ml of MS broth and incubated at 28±2°C overnight with shaking. Overnight cultures (4 ml) were inoculated into 36 ml of MS broth and incubated overnight as before. Cultures were centrifuged at 3000 *g* for 5 min, resuspended in 9 ml of ultrapure type I water (ThermoScientific Barnstead E-pure), stored in the dark at room temperature, and monitored for a period of two weeks. Three independent replicates per strain were conducted for statistical analysis. At day 1, day 7 and day 14, an aliquot from each of the 12 tubes (4 strains × 3 replicates) was taken for i) colony forming unit (CFU) counts, ii) light microscopy, and iii) scanning electron microscopy (SEM) (see below).

### Ultrastructural analysis

Changes in morphology were monitored periodically using light microscopy, SEM, and transmission electron microscopy (TEM). For light microscopy, cells (5 μl of culture) were air dried on a microscope glass slide, stained with safranin and observed using a Leica DM2500 with differential interference contrast (Leica Microsystems, USA). For SEM, cells (5 μl of culture) were fixed in 2.5% glutaraldehyde (v/v) at 4°C overnight. Samples were filtered through Isopore™ membrane (0.2 μm GTBP) (Millipore, USA), dehydrated in a graded ethanol series (50%, 70%, 90% and 100%), critical-point dried in CO_2_ in an EMS 850 (Electron Microscopy Science, USA) and coated with gold palladium alloy in an EMS 550X (Electron Microscopy Science). The coated samples were examined using a Zeiss EVO 50 (Zeiss, Germany). Ten microscope fields, at 3000X magnification, were randomly taken of each isolate on each sampling day. The percentage of coiled forms and bacillus were determined by counting all the cells present in each field. In addition, the average length of 10 randomly selected cells per field was measured.

For TEM, 250 μl of culture were fixed in 0.1 M PBS, pH 7.2 containing 2.5% glutaraldehyde, and 2% formaldehyde. After 90 min at room temperature, cells were washed in PBS and fixed in 1% OsO_4_ for another 90 min prior to dehydration in a graded ethanol series (30-100%), washed in propylene oxide (PO) and infiltrated in epoxy resin (EMbed 812, Electron Microscopy Sciences, Pennsylvania, USA) following manufacturer’s instructions for soft block hardness replacing 3:1 PO:Resin mix, 1:3 PO:Resin mix, 1:3 PO:Resin mix, resin washes and polymerized. After microtoming, samples were observed using a Zeiss EM 10C 10CR Transmission Electron Microscope (Zeiss, Germany).

### Viability of coiled cells

To prove that the coiled forms were viable and not degenerative forms, a ‘dilution to extinction’ strategy was used. Cultures from the 14 day microcosm experiment were 10-fold diluted in MS broth until 10^-13^ and incubated for 48 h at 28±2°C. If tubes showed turbidity then, 100 μl was inoculated onto MS agar in triplicate and typical *F*. *columnare* colonies were annotated. To further evaluate the survival potential of starved cells, strain ALG-00-530 was selected to determine the membrane integrity of starved versus non-starved cells. Fresh (24 h) and starved (1-month, 3-month, and 5-month) cultures of ALG-00-530 were used for this experiment. Starved cultures were prepared as described before. Membrane potential was estimated with LIVE/DEAD BacLight Bacterial Viability Kit (Invitrogen, USA) following manufacturer’s instructions (SYTO 9 and propidium iodine were mixed 1:1 before adding to the cultures). Stained cells were observed under a Zeiss epifluorescent microscope (Zeiss, Germany) using appropriate filters. Green (live) and red (dead) cells from 10 microscope fields were photographed and counted at 400X.

### Virulence of the coiled forms

To test the virulence potential of the starved cells in channel catfish, we challenged channel catfish with fresh ALG-00-530 and 2 week-old starved cultures. Challenge protocols have been described previously in detail [19]. Briefly, challenge experiment consisted of three treatments: fresh (24 h) ALG-00-530, 2 week-old ALG-530, and unchallenged control. Each treatment consisted of three randomized replicates (tanks) containing 10 channel catfish per tank (mean weight: 0.8±0.1 g; mean leght 4.5±0.5 cm). Fish were challenged by immersion in a bath containing 2.5×10^7^ and 1.9×10^6^ CFU/ml of the fresh and 2-weeks old ALG-00-530, respectively. Controls were exposed to MS broth without bacteria. Fish were monitored at 12 h intervals for abnormal behavior, loss of appetite and mortality. Moribund fish were sampled for *F*. *columnare* and putative colonies were confirmed using following standard protocols [20].

### Growth curve

To compare the growth potential of fresh and starved cultures 20 μl of a 24 h, 1-month, and 3-month-old cultures of strain ALG-00-530 (obtained as described above) were inoculated into microtiter plates containing fresh MS medium (80 μl) and allowed to grow at 28±2°C for 24 h. Cell optical density (OD_595_) was measured at regular intervals using a Synergy HT microplate reader (Bio-TEK, USA). Immediately after each reading, 100 μl of the LIVE/DEAD mixed dyes were added to each well and fluorescence was quantified at 528 nm (green) and 590 nm (red). Four independent replicates were carried out per culture.

### Revival of starved cultures

To better understand how the starved cells transitioned into a rich-nutrient environment, we monitor the ultrastructural changes in five-month old ALG-00-530 cultures when they were exposed to different levels of nutrients present in MS medium. Starved cells were inoculated (1:100 dilution) into the following media: MS, 10 times diluted MS (MS-10), MS containing salts and tryptone but not yeast extract (MS-T), MS containing salts and yeast extract but not tryptone (MS-Y), and MS containing salts but not organic nutrient (MS-S). The experiment was carried out in triplicate. Tubes were incubated at 28°C with gentle shaking for 78 h. Cell morphology was analyzed at regular intervals by using light microscopy and SEM as previously described. Cell optical density (OD_595_) was measured as proxy for bacterial growth (see above).

### Statistical analysis

Colony forming unit counts were converted to base 10 logarithms to fit the model assumption of normal distribution. One-way analysis of variance (ANOVA) was used to determine the differences in *F*. *columnare* CFU/ml from the short-term survival study. Welch’s ANOVA (allowing for unequal variance) was used to determine differences of bacillus *versus* ‘coiled’ forms. If either ANOVA or Welch’s ANOVA was statistically significant (*P* value < 0.05), Tukey’s method and Scheffe’s method were applied to perform post hoc, pair-wise comparisons at α = 0.05 for the means of log *F*. *columnare* counts or the Dunnett’s T3 test (allowing unequal variance) as post hoc, pair-wise comparisons for ‘bacilli/coiled’ forms at α = 0.05. Mortality data were compared by ANOVA using the Duncan’s multiple range test. Calculations were done using the OriginPro version 8.5 (OriginLab Co., Northampton, MA).

## Results

### Survival under starvation conditions

Table 1 shows the culturability of the four *F*. *columnare* strains when subjected to two weeks of starvation conditions in ultrapure water. Initial numbers (>10^9^ CFU/ml) were statistically identical in all strains but the culturability loss rate varied among strains with significant differences found during the 14 day period. Colony forming units in ATCC 23643 strain dropped from 4.8×10^9^ CFU/ml to 3.2×10^5^ CFU/ml at day 7 and down to 7.9×10^4^ CFU/ml at day 14. In strain ARS-1, a 2-log statistically significant reduction in culturability was observed at day 7 but CFU/ml did not significantly change at day 14. Strain ALG-00-530 maintained similar CFU/ml at day 1 and 7 but a significant 3-log reduction was observed at day 14. Strain AL-02-36 showed significant CFU/ml reductions at day 7 (a near 3-log decrease) and day 14 (final count of 3.4×10^5^ CFU/ml). Colony forming units were significantly lower at day 14 than at day 1 in all strains. Genomovar I strains (ATCC 23643 and ARS-1) yielded the lowest and highest numbers of viable cells at day 14, respectively; thus, no correlation could be inferred between cell survival and genomovar ascription.

**Table 1:** Total number of colony forming units per ml (mean ± standard error) obtained when cells were maintained in ultrapure water

**Time**	**ATCC 23643**	**ARS-1**	**ALG-00-530**	**ALG-02-36**
Day 1	9.687 ± 0.135 ^a,w^	9.929 ± 0.040 ^a,w^	9.743 ± 0.004 ^a,w^	9.507 ± 0.060 ^a,w^
Day 7	5.556 ± 0.024 ^b,w^	7.717 ± 0.414 ^b,x^	9.688 ± 0.135 ^a,y^	6.895 ± 0.021 ^b,z^
Day 14	4.908 ± 0.568 ^c,w^	7.451 ± 0.080 ^b,x^	6.732 ± 0.060 ^b,y^	5.533 ± 0.420 ^c,w^

Data was log 10 transformed to ensure normality. Significantly different means (P < 0.05) within columns are noted with superscripts a, b, and c. Superscripts w, x, y and z denote significantly different means (P < 0.05) within rows.

### Ultrastructural changes under starvation conditions

**Figure 1:**
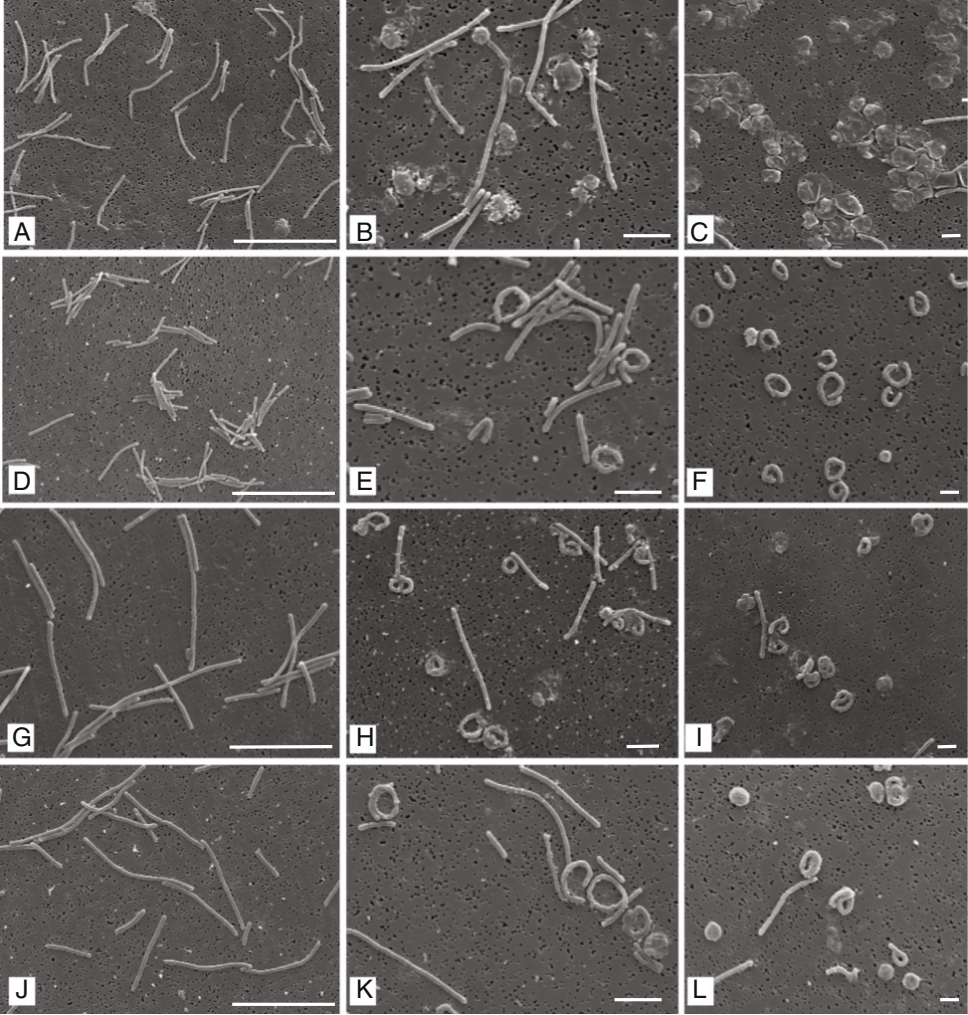
**Morphology of *Flavobacterium columnare *cells during starvation in ultrapure water as determined by SEM.** Panels **A**, **B**, and **C** display ATCC 23643 strain. Panels **D**, **E**, and **F** show ARS-1 strain. Panels **G**, **H**, **I** show ALG-00-530 strain. Panels **J**, **K**, and **L** display ALG-02-36 strain. Panels **A**, **D**, **G**, and **J** show cells at day 1 (scale bar 10 μm); panels **B**, **E**, **H**, and **K** display 7 days starved cells (scale bar 5 μm); panels **C**, **F**, **I**, and **L** show 14 day starved cells (scale bar 1 μm).

Samples were collected at day 1, 7 and 14 during the short-term starvation experiment and examined using light microscopy (see Additional file 1: Figure S1.1) and SEM. Figure 1 shows the evolution of *F*. *columnare* morphological changes in all four strains during 14 days of starvation in ultrapure water examined by SEM. In all strains, long and thin bacilli characteristic of the species *F*. *columnare* were observed at day 1 although significant differences in length were noted among strains. Strains ATCC 23643 and ALG-00-530 measured 6.61±0.4 μm and 6.11±0.5 μm, respectively (mean of 10 bacilli) and were not significantly different. However, ARS-1 cells were significantly shorter with a mean length of 5.05±0.1 μm. Conversely, strain ALG-02-36 cells were the longest at 7.32±0.6 μm. At day 7, the morphology of the cells had drastically changed with approximately half of the rods adopting a curled form; some forming circles while others adopted a coiled conformation. In strain ATCC 23643, coiled rods were covered by an extracellular matrix (Figure 1B). By day 14, only a few bacilli remained as straight rods while the vast majority of the cells had adopted a coiled conformation.

**Figure 2:**
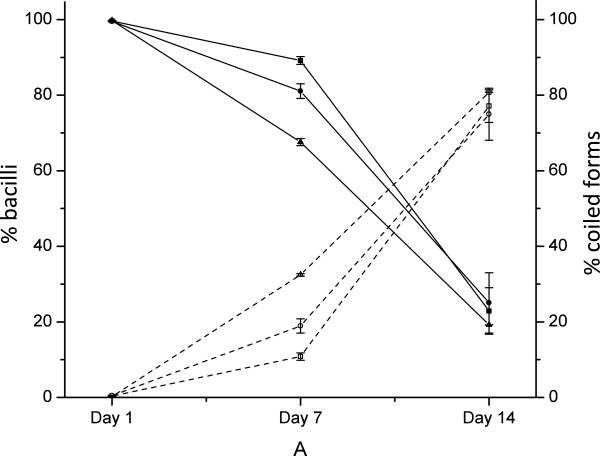
**Percent of bacillus and coiled forms observed over time during starvation in ultrapure water.** Bacillus and coiled forms are represented by solid and open symbols, respectively. ARS-1 (■), ALG-00-530 (●), and ALG-02-36 (▲).

Figure 2 shows how the cell morphology shifted from long and thin rods to coiled forms at 14 days. Data on ATCC 23643 strain could not be analyzed due to the matrix that covered the cells making morphotype ascription unfeasible. At day 1, there were not significant differences between mean percent of bacillus forms observed in ARS-1, ALG-00-530, and ALG-02-36 strains. At day 7, the percent of bacillus forms in ALG-00-530 was significantly lower than in the other two strains. At day 14, 75% or more of all observed cells were coiled forms in all strains. The number of coiled forms at day 14 was statistically identical in all three strains.

**Figure 3:**
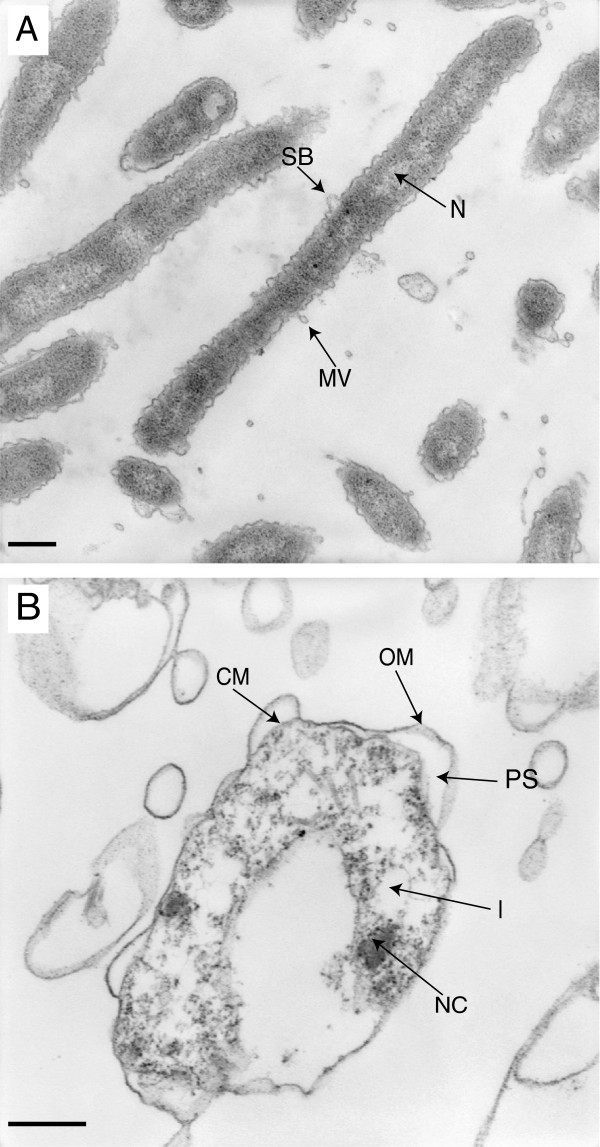
**TEM observations of *Flavobacterium columnare *ALG-00-530 strain in ultrapure water.** Panel **A**, day 1 after transfer to ultrapure water. Panel **B**, maintained in ultrapure water for 150 days. Arrows indicate surface blebbing (SB), membrane vesicle (MV), nucleoid (N), cell membrane (CM), outer membrane (OM), periplasmic space (PS), inclusion (I), and nucleoid compaction areas (NC). Scale bars represent 500 nm.

The ultrastructure of *F*. *columnare* under starvation was further investigated using TEM. At day 1, the ultrastructure of ALG-00-530 shows the outer membrane of the cells with formations that appear to be membrane vesicles breaking off the cells (Figure 3A). No clear glycocalyx or capsule was detected in any cell. Fine-granular cytoplasmatic structure and a denser area that typically corresponds with the nucleoid were observed. By contrast, cells starved for 14 days showed greater heterogeneity in their structure with many apparently empty membrane vesicles and lysed cells (Figure 3B). The remaining structurally intact cells were curved (some were coiled) and were characterized by an enlarged periplasmic space, a fine granular structure in the periplasm that lack any clearly visible ribosomes, regions of nucleoid compaction (electron-dense areas), and some inclusions.

### Viability of coiled cells

**Figure 4:**
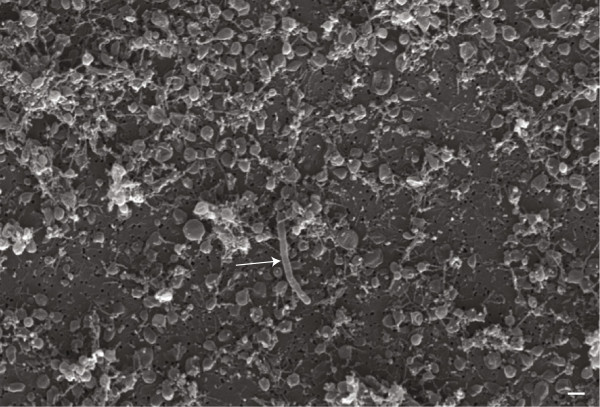
***Flavobacterium columnare *ALG-00-530 strain after starvation in ultrapure water for 150 days as determined by SEM.** Arrow indicates the only bacillus observed in this preparation. Scale bar represents 1 μm.

By using a ‘dilution to extinction’ strategy, the few bacilli that remained in the microcosm after 14 days of starvation were diluted out until, by probability, all cells present in the dilutions were coiled. Dilutions up to 10^-8^ yielded positive tubes (three independent dilution experiments were carried out per strain) in all cultures. Based on the CFU/ml value calculated by plate count and with more than 80% of cells adopting the coiled form (as per SEM observations), the observed growth in the 10^-8^ dilution tubes was due to the coiled cells ability to grow and multiply in broth. The notion that the coiled forms were indeed viable was further tested using ALG-00-530 cultures maintained in ultrapure water for up to 5 months. In this culture, more than 99% of cells visible under SEM were coiled at 5 months (Figure 4). After dilution to extinction, 5-month old ALG-00-530 cells were able to grow in broth after all bacilli cells had been diluted out. Interestingly, aged ALG-00-530 cells were covered by a matrix similar to that observed in 14-day old ATCC 23643 cells (Figure 1C). In addition, cells were connected by what appeared to be fimbriae like structures that were not observed in 14 day old cultures.

### Virulence of starved cells

Channel catfish challenged with 24-h old ALG-00-530 started to display signs of columnaris disease at 12 h post-challenge. First mortalities in that group were observed within 24 h of exposure to the pathogen and reached 100% mortality at 48 h post-challenge. *Flavobacterium columnare* was isolated from all dead fish. Conversely, fish challenged with 2-weeks old ALG-00-530 did not show any signs of columnaris disease and *F*. *columnare* was not recovered from any fish analyzed (upon experiment completion 10% of the challenged fish were necropsied). No mortalities were observed in the control group. These results showed that starved cells of *F*. *columnare* are avirulent for channel catfish under our experimental challenge conditions.

### Growth curves

**Figure 5:**
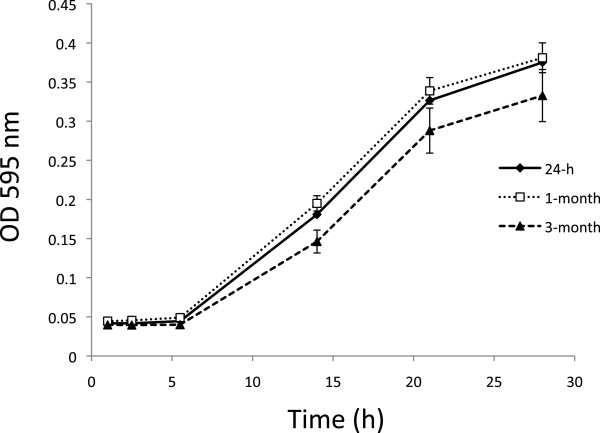
**Growth curves of 24-h (♦), 1-month (□), and 3-month (**^**♦**^**) old cultures of strain ALG-00-530 cultivated in MS at 28°C.** Data points represent means and error bars represent standard errors.

To compare the viability of cells present in fresh cultures with those from starved cultures, we monitored the growth patterns of fresh and starved cultures of strain ALG-00-530. Figure 5 shows the growth curve of 24 h, 1-month, and 3-month old cultures. Initial optical densities were adjusted in all three cultures and were not statistically significant. Both growth curves from 24-h and 1-month old cultures were statistically identical. The 3-month old culture showed a slightly but statistically significant reduced growth after 15-h post inoculation. The growth curves data showed that the viability of the starved cells is maintained but a significant decrease in cell fitness was observed at 3-months.

Cells were also monitored using the ratio between the LIVE/DEAD dyes over time (same sampling times as shown in Figure 5), but no significant difference between all three cultures was observed throughout the time course (data not shown). Microscopy observation of cells stained with the LIVE/DEAD kit yielded inconsistent results. Propidium iodide stained the majority of both coiled cells and rods even when fresh cultures (24 h old) were used. After many repeats, we hypothesized that slight manipulations (ie, centrifugation or osmotic shock) of the cells may damage cell membranes thus allowing the propidium iodine to penetrate into the cells.

### Revival of starved cultures

**Figure 6:**
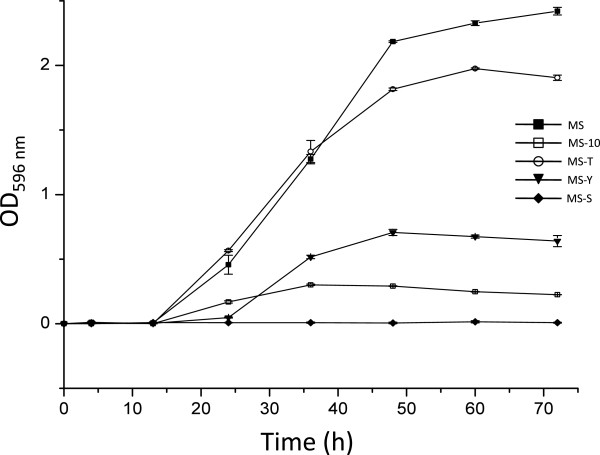
**Growth curves of 5-month old *Flavobacterium columnare *ALG-00-530 cultures incubated under different nutrient conditions.** Modified Sheih (MS) medium (■), diluted MS (MS-10) (□), MS without yeast extract (MS-T) (○), MS without tryptone (MS-Y) (**♦**), and MS without nutrients (MS-S) (▼). Data points represent means and error bars represent standard errors.

The growth curves of 5-month old ALG-00-530 inoculated into media with different nutrient loads are shown in Figure 6. Cell cultured in MS broth reached the highest cell density followed by cells cultured in MS-T (no yeast extract). MS-Y broth supported cell growth but at much higher levels than MS and MS-T and the lag phase was noticeable longer in this medium. Diluted MS (MD-10) produced the lowest cell density. No growth was observed in broth without nutrients (MS-S). The lag phase extended up to 12 h post-inoculation (except for MS-Y which lasted 24 h) and significant differences in ODs were observed between MS&MS-T and MS-10&MS-Y at 24 h. Cell densities became statistically significant between all culture media after 48 h post inoculation and remained different until the end of the experiment.

**Figure 7:**
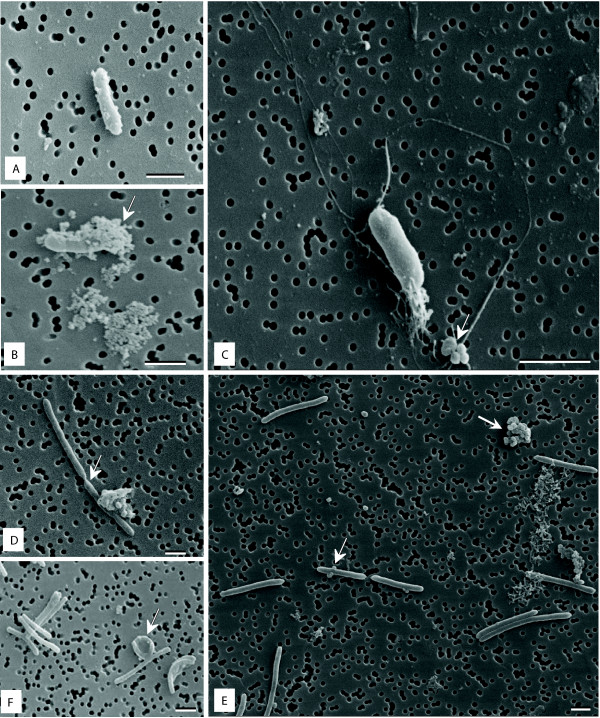
**Morphology changes of *Flavobacterium columnare *starved cells during revival in different nutrient media.** Panels **A** and **B**, cells cultured in Modified Sheih (MS) medium at 4 h post-inoculation (arrows point to small membrane vesicles). Panel **C**, a cell cultured in diluted MS (MS-10) at 4 h post-inoculation (arrow indicates fimbriae). Panel **D**, active cells division observed in MS-10 cultures at 12 h post-inoculation. Panel **E**, cells actively growing in MS at 36 h post-inoculation displaying membrane vesicles (arrow). Panel **F**, coiled forms (arrow) observed in MS-10 cultures at 36 h post-inoculation. Scale bars represent 1 μm.

To determine what morphological changes, if any, accompanied the revival of starved cells under different nutrient conditions, we examined the cell morphology at 4, 12, and 24 h post-inoculation using both light microscopy (data not shown) and SEM (Figure 7). Morphology of starved cells at time 0 (prior inoculation) was similar to that displayed in Figure 5. At 4 h post-inoculation, cells were scarce in all media and appeared as short rods (1–2 μm). In MS broth and MS-10, cells were covered by small spheres that in some instances (Figure 7A, B) coated most of the cell surface. This spheres resembled membrane vesicles that could derive from the external cell membrane of the cells. We did not observe any coiled forms at this time. Some cells cultured in MS-10 exhibited long fimbrie and this was not detected in any of the other media (Figure 7C). The presence of these structures may explained why at 4 h post-inoculation into MS-10, cells appeared as tight clusters under light microscopy (data not shown). At 12 h, cell become more elongated and cell division was observed in MS (Figure 7D) and MS-T. Cells reached the average size previously observed for ALG-00-530 strain after 24 h of incubation in MS and MS-T. Between 24 and 36 h post-inoculation, we observed the production of what appeared to be surface blebbing leading to membrane vesicle formation in all examined cultures (Figure 7E). These vesicles were much larger than those observed at 4–12 h (Figure 7A-C). At 48 h all cells have recovered the typical morphology of ALG-00-530 cells in the exponential phase and resemble that observed in Figure 1 (G). Morphological changes between cells cultured in MS, MS-10, MS-T, and MS-Y were not different. Interestingly, and at 36 h, we observed the appearance on coiled cells in MS-10 broth (Figure 7F) suggesting that those cells had utilized all available nutrients and were entering the starvation phase.

## Discussion

It is widely accepted that most bacteria encounter low nutrient conditions during their life cycles and that adaptation strategies must be in place to survive those adverse conditions. Starvation-induced activities include differentiation into resistant forms that maintain viability in absence of nutrients [21]. Some of the resistant forms that bacteria can differentiate into include spores, ultramicrobacteria and viable but not culturable (VBNC) cells [22]. A common denominator in bacteria subjected to starvation is the ‘rounding up’ phenomenon by which cells become rounder, adopting a coccus shape morphology [22]. In addition, starved cells tend to show a reduction in size and therefore an increase in their surface-to-volume ratio, which may facilitate the uptake of substrates from a nutrient-poor environment. Our study showed that *F*. *columnare* develops a very unique cell configuration when subjected to starvation characterized by ring or coiled forms that, overtime, developed an envelope layer. Cells maintained their length but their overall shape changed from long and thin bacilli to round forms by curving over themselves. The strategy adopted by *F*. *columnare* did not increase the surface-to-volume ratio of the cell but reduced the surface exposed to the elements. The secretion of amorphous extracellular polysaccharides have been described in other Gram negative bacteria and data suggest they conferred protection against osmotic and oxidative stresses during starvation [22]. If the matrix that was observed around the *F*. *columnare* starved cells in the later stages was indeed secreted to provide protection against starvation or unfavorable environments then, the phenomenon of ‘coiling’ could be considered a starvation-induced activity since it would allow the cells to save energy by producing less of the protective envelope to cover themselves.

The presence of coiled or curved cells in old *F*. *columnare* cultures was first reported by Garnjobst [23] in 1945 who described those cells as degenerative since the author failed to recover colonies after passing them onto fresh medium. Since then, the presence of spheroplasts or degenerative forms have been reported in several *Flavobacterium* species [24]. Garnjobst [23] described how those cells, in their latter stages, were covered by a ‘veil of secreted slime’ that make the ‘coiled’ or ‘ring’ cells appeared as coccus-shaped cells. Her descriptions matched our observation precisely, both based on light-microscopy (see Additional file 1: Figure S2) and SEM (Figure 2) but our results showed that the ‘coiled’ forms are not degenerative but viable and culturable after at least one month of starvation. This was proven by comparing the growth curves between fresh and 1-month starved cultures in where no differences were observed. If starved cells were degenerative forms and observed growth was due to the few remaining bacilli observed then, a significant lag phase should be observed in cultures with a predominant population of coiled forms. The main difference between her study and ours is that, Garnjobst [23] aged *F*. *columnare* cultures in high nutrient solid medium while we maintained our cultures in liquid and in absence of any organic nutrient. Excess of toxic metabolites and oxygen radicals in agar plates could explain the differences observed in culturability of aged *F*. *columnare* cells.

When starved cells were exposed to a different range of nutrients, their morphology transitioned from coiled forms to short bacilli. We failed to observe the cells ‘uncoiling’ but they morphed into noticeable smaller cells rather quickly. Cells exposed to nutrients produced numerous membrane vesicles that seem to be secreted into the medium thus reducing the overall volume of the cells. After this transition phase in where the cells reduce their volume and recovered their rod morphology, cells started to actively divide as confirmed by a parallel increase in cells numbers (SEM) and cell density values. Nutrients clearly reversed the structural changes induced during starvation. From our experiments, we conclude that *F*. *columnare* ‘coiled’ forms are viable but do not reproduce unless they revert back to the rod morphology.

Survival under long-term starvation conditions in freshwater has also been demonstrated in the close species *F*. *psychrophilum*[14,25]. However, the morphological changes observed in *F*. *psychrophilum* during starvation were less dramatic than those observed in *F*. *columnare*[14]. Few cells adopted a ‘ring-type’ structure but the main distinctive characteristic of starved *F*. *psychrophilum* cells was the formation of enlarged areas along the length of the cells or at one of the ends. SEM images of *F*. *psychrophilum* starved cells did not show the matrix layer covering the cells that we observed in *F*. *columnare*. Nevertheless, ultrastructural similarities were found between these two species. Surface blebbing and membrane vesicle formation was observed in fresh cultures of *F*. *columnare* and during the revival process of starved cells similar to those reported in *F*. *psychrophilum*[26]. Although the role of bleb formation and release of membrane vesicles is not clear, it has been postulated they may play a role in host-pathogen interaction due to the high content of antigenic proteins present in *F*. *psychrophilum* membrane vesicles. Further studies on the role that these ultrastructures may play in *F*. *columnare* pathogenesis are needed. The typical capsule described for *F*. *columnare*[5] and *F*. *psychrophilum*[14] was missing from our TEM images probably due to different sample preparation methods. It is likely that during sample preparation for TEM, the capsule or mucus layer observed by SEM was removed since we did not use a capsule stabilization protocol.

Differences in cell culturability were observed between strains although those could not be correlated with their genetic group. The strains used in this study were choosen based on their genotype and source of isolation [15]. Strains ARS-1, ALG-00-530 and AL-02-36 behaved similarly throughout the experiment and the numbers of coiled forms at 14 days were statistically identical. The initial length of the cells seemed not to influence the coiling process since both the shortest (ARS-1) and the longest (ALG-02-36) strains behaved similarly. In the type strain ATCC 23643, coiled cells were covered by a matrix layer that made difficult to observe the cell structure in detail. SEM observations of starved ATCC 23643 cells resembled those described in starved *Vibrio cholerae* cells by Chaiyanan et al. [27] in where *V*. *cholerae* cells had remained viable for a 2-year period. The appearance of coiled cells covered by a matrix was also observed in strain ALG-00-530 after 5 months in ultrapure water. Cells were connected by what appeared to be fimbriae, a characteristic structure that has also been reported in other long-term starvation studies [13,27,28]. Our results showed that strains of *F*. *columnare* followed a similar strategy to survive under lack on nutrients by adopting a coiled conformation and secreting a matrix layer, although this process occurred faster in some strains.

Under starvation conditions and in absence of organic nutrients, *F*. *columnare* can survive for at least 5 months at ambient temperature in sterile water. In a previous study [10], the authors proposed that *F*. *columnare* survived the nutrient-deprived conditions by utilizing nutrients released from dead cells that allowed cultures to maintain constant growth over time. Our results contradict this assumption because in all our microscopic observations we failed to detect any cells undergoing cell division although we did note some lysed cells in our cell preparations that likely released nutrients into the medium. Based on our data, and at 5 months under starvation, more than 99% of the *F*. *columnare* cells underwent a dramatic change in morphology and cell structure into what can only be considered dormant or resistant forms. This behavior is typical of copiotrophic bacteria that can survive under oligotrophic conditions but without active reproduction [21]. Moreover, 3-month old *F*. *columnare* cells were not able to outcompete with young cells when provided with nutrients which indicates *F*. *columnare* lose fitness overtime when subjected to starvation conditions.

The new observations presented in this study demonstrate a unique state in the *F*. *columnare* life cycle induced by starvation. This state (coiled form) should not be regarded as degenerative but an active adaptation to lack of nutrients allowing *F*. *columnare* to remain viable in water, in absence of organic matter, and even without salts for an extended period of time. This bacterium is likely to encounter starvation conditions after nutrients provided by the host are exhausted and bacterial cells are released back into the water column. This stage in the life cycle of *F*. *columnare* indicates that water can act as reservoir and served as dispersant mechanism for this pathogen. However, *F*. *columnare* should not be considered a facultative oligotroph since no cell replication was observed under very limited nutrient content (originated from lysed cells) suggesting that water is a transient environment for this bacterium. Furthermore, starved cells failed to infect channel catfish thus low organic waters should not be considered the primary reservoir for this pathogen. The notion that *F*. *columnare* may have a restrictive ecological niche is supported by the recently published complete genome of *F*. *columnare* that predicts a lifestyle in close association with its host [29]. However, further studies on the biology of *F*. *columnare* are required to fully understand its life cycle.

## Conclusion

Our results showed that *F*. *columnare* responds to starvation by adopting a coiled conformation instead of using a ‘rounding up’ strategy. These coiled cells remained culturable over time although prolonged starvation seemed to decrease cell fitness and resulted in loss of virulence. Our data show that *F*. *columnare* induces a long-term survival response mechanism upon encountering adverse conditions that is reversed when the bacterium is provided with appropriate nutrients.

## Competing interests

The authors declare that they have no competing interests.

## Authors' contributions

CA, conceptual and experimental design and data analysis and interpretation; SLF, light microscopy, scanning microscopy of starved cells, transmission electron microscopy, and growth curves; WC, scanning microscopy of revived cultures and virulence studies; OOF, growth curves of starved cultures. All authors approved the final manuscript.

## Supplementary Material

Additional file 1 **Figure S1.** Morphology of *Flavobacterium columnare* cells during starvation in ultrapure water as determined by light microscopy. Panels A, B, and C display ATCC 23643 strain. Panels D, E, and F show ARS-1 strain. Panels G, H, I show ALG-00-530 strain. Panels J, K, and L display ALG-02-36 strain. Panels A, D, G, and J show cells at day 1; panels B, E, H, and K display 7 days starved cells; panels C, F, I, and L show 14 day starved cells. Scale bars represent 25 μm. Characteristic coiled forms are noted by arrows. Click here for file
