# Supplementary figures and images for: Adaptive response to starvation in the fish pathogen Flavobacterium columnare: cell viability and ultrastructural changes
Source: BMC Microbiol. 2012 Nov 19;12:266. doi: 10.1186/1471-2180-12-266 (PMC3517764; doi:10.1186/1471-2180-12-266)

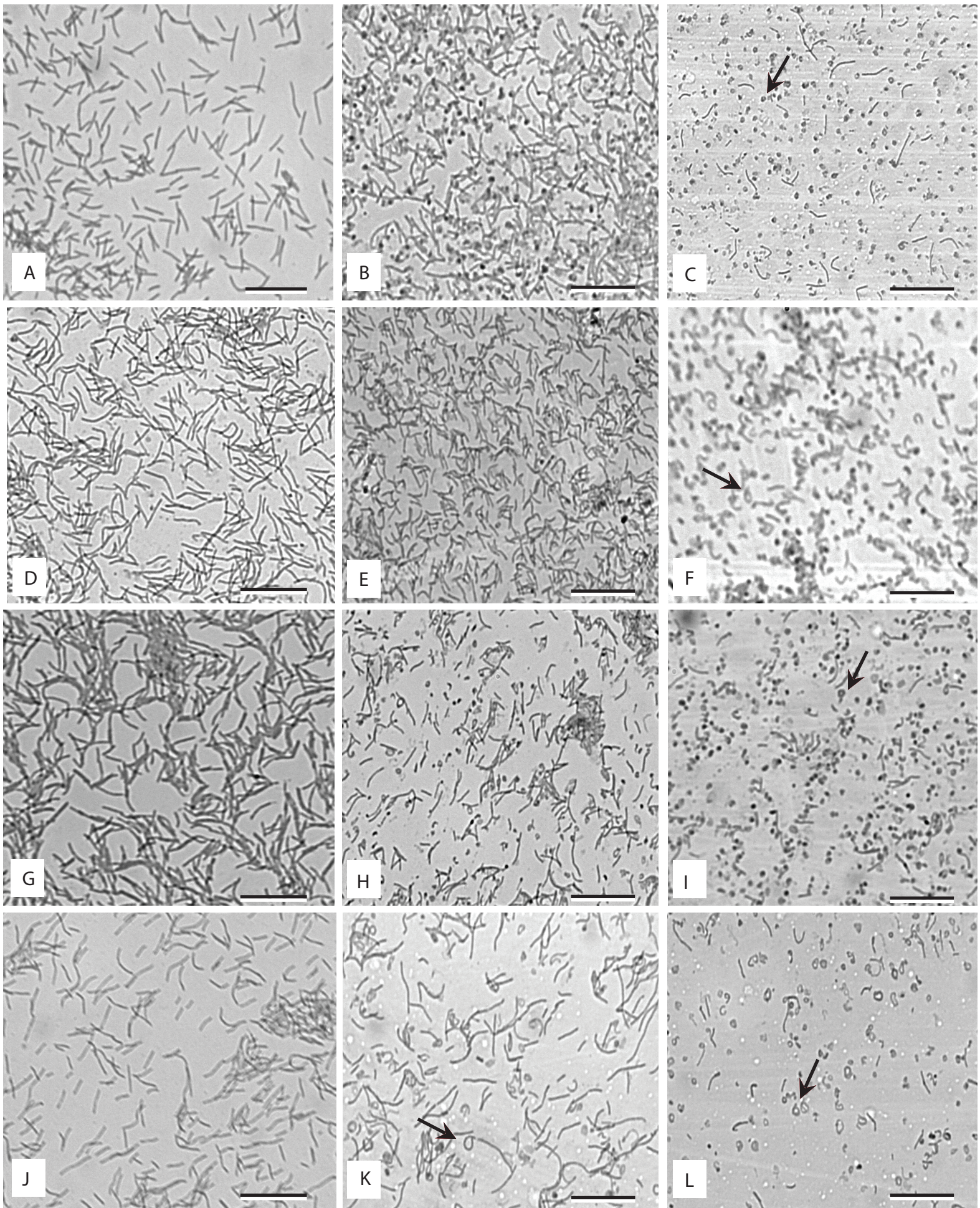

Figure 2S. LaFrentz, Cai, Olivares & Arias

Supplement: Additional file 1 — Figure S1. Morphology of Flavobacterium columnare cells during starvation in ultrapure water as determined by light microscopy. Panels A, B, and C display ATCC 23643 strain. Panels D, E, and F show ARS-1 strain. Panels G, H, I show ALG-00-530 strain. Panels J, K, and L display ALG-02-36 strain. Panels A, D, G, and J show cells at day 1; panels B, E, H, and K display 7 days starved cells; panels C, F, I, and L show 14 day starved cells. Scale bars represent 25 μm. Characteristic coiled forms are noted by arrows. [file 1471-2180-12-266-S1.pdf]
